# Health workers’ perceptions of facilitators of and barriers to institutional delivery in Tigray, Northern Ethiopia

**DOI:** 10.1186/1471-2393-14-137

**Published:** 2014-04-10

**Authors:** Tesfay Gebrehiwot, Miguel San Sebastian, Kerstin Edin, Isabel Goicolea

**Affiliations:** 1 Department of Public Health, College of Health Sciences, Mekelle University, Mekelle, Ethiopia; 2 Department of Public Health and Clinical Medicine, Epidemiology and Global Health, Umeå University, Umeå, Sweden; 3 Department of Nursing, Umeå University, Umeå, Sweden

**Keywords:** Health workers, Perception, Institutional delivery, Health extension programme, Health facilities, Barriers, Ethiopia

## Abstract

**Background:**

Evidence shows that the three delays, delay in 1) deciding to seek medical care, 2) reaching health facilities and 3) receiving adequate obstetric care, are still contributing to maternal deaths in low-income countries. Ethiopia is a major contributor to the worldwide death toll of mothers with a maternal mortality ratio of 676 per 100,000 live births. The Ethiopian Ministry of Health launched a community-based health-care system in 2003, the Health Extension Programme (HEP), to tackle maternal mortality. Despite strong efforts, universal access to services remains limited, particularly skilled delivery attendance. With the help of ‘the three delays’ framework, this study explores health-service providers’ perceptions of facilitators and barriers to the utilization of institutional delivery in Tigray, a northern region of Ethiopia.

**Methods:**

Twelve in-depth interviews were carried out with eight health extension workers (HEWs) and four midwives. Each interview lasted between 90 and 120 minutes. Data were analysed through a thematic analysis approach.

**Results:**

Three themes emerged from the analysis: the struggle between tradition and newly acquired knowledge, community willingness to deal with geographical barriers, and striving to do a good job with insufficient resources. These themes represent the three steps in the path towards receiving adequate institutional delivery care at a health facility. Of the themes, ‘increased community awareness’, ‘organization of the community’ and ‘hospital with specialized staff’ were recognized as facilitators. On the other hand, ‘delivery as a natural event’, ‘cultural tradition and rituals’, ‘inaccessible transport’, ‘unmet community expectation’ and ‘shortage of skilled human resources’ were represented as barriers to institutional delivery.

**Conclusions:**

The participants in this study gave emphasis to the major barriers to institutional delivery that are closely connected with the three delays model. Despite the initiatives being implemented by the Tigray Regional Health Bureau, much is still needed to enhance the humanization approach of delivery care on a broader level of the region. A quick solution is needed to address the major issue of lack of transport accessibility. The poor capacity of the HEWs to provide delivery services, calls for reconsidering staffing patterns of remote health posts and readdressing the issue of downgraded health facilities would address unmet community needs.

## Background

In 2000 at the United Nations (UN) Millennium Summit, 147 heads of states adopted the Millennium Declaration, with the aim, among others, of reducing worldwide maternal mortality by 2/3 by 2015. The deadline is approaching and the progress to reduce maternal mortality has been slow. This is particularly worrying in sub-Saharan Africa where more than 162,000 women die each year during pregnancy and childbirth, most of them because they lack access to maternal health care, mainly skilled delivery attendance, comprehensive and basic emergency obstetric and neonatal care [1-5].

Maternal health, and in particular access to skilled birth attendants, is highly stratified by poverty and other social determinants of health [6]. In 1994, Thaddeus and Maine proposed a model to explain the social causes of maternal deaths, beyond the already known medical causes (e.g. haemorrhage, obstructed labour, sepsis). They described how maternal deaths occurred primarily due to three types of delays in accessing health-care services that are able to deal with obstetric complications: 1) delay when deciding to seek appropriate medical care, 2) delay when reaching an appropriate obstetric facility, and 3) delay when receiving adequate care once the facility is reached [7]. ‘The three delays model’ aimed not only to classify in which of these steps maternal complications and deaths took place, but also to explore ways to prevent deaths by minimizing such delays.

Almost 20 years later, evidence shows that these same three delays are still contributing to maternal deaths in low-income countries [2,8]. Poor birth preparedness, geographical inaccessibility, institutional delivery not thought as necessary, family influence on the decision-making process, unmet needs for community-based care in obstetric emergencies and fear of hospital settings are common factors related to the first type of delay [9-11]. Late and/or poor-quality referral, transport not available and inadequate decisions by husband/relatives have been associated with the second type of delay [9,10,12]. Finally, lack of supplies and staff, poor quality of care and multiple delays due to second referrals have been reported in the literature as part of the third type of delay [9-12]. To promote maternal health and to prevent unnecessary suffering and deaths, it is important to recognize and understand the factors involved in these delays.

### Maternal health care in Ethiopia

With a maternal mortality ratio of 676 per 100,000 live births and 19,000 maternal deaths annually, Ethiopia is a major contributor to the worldwide death toll of mothers [13]. The major immediate causes of maternal deaths in Ethiopia are infections/sepsis (47.1%), haemorrhage (29.4%), severe pre-eclampsia/eclampsia (7.6%), obstructed/prolonged labour and ruptured uterus (2.9%), with complications from unsafe abortion accounting for the remaining 2.9% of maternal deaths. The indirect obstetric causes are anaemia, HIV/AIDS and cardiovascular diseases and account for 10% of maternal deaths [14]. The major supply side constraints that contribute to maternal death are shortages of skilled midwives, weak referral systems at health centres, and inadequate availability of basic and comprehensive emergency obstetric and neonatal care equipment. On the demand side, cultural and societal norms, distances to functioning health centres and financial barriers are considered chief constraints; this is similar in other low-income countries [15].

In response to the slow progress on tackling maternal mortality, the Ethiopian Ministry of Health launched a community-based health-care system in 2003, the Health Extension Programme (HEP), rooted in a primary health-care approach [16]. The HEP is designed to improve equitable access for preventive essential health interventions through community-based health services and to achieve significant basic health-care coverage through the provision of a staffed health post to serve an area of approximately 3,000 to 5,000 people – a *kebele*, the lowest administrative unit. Each *kebele* has one health post where two health extension workers (HEWs), after completion of one year’s training, are employed to provide preventive, basic curative health services to the community and carry out activities related to health promotion [17]. The HEP has been implemented throughout Ethiopia, with more than 33,000 HEWs already trained and deployed since 2004 [18].

Key maternal and child health services are free of charge through the health-care financing strategy; moreover, the Federal Ministry of Health (FMOH) has redesigned a three-tier health-care delivery system: 1) the district primary hospitals (to cover 60,000–100,000 people), 2) health centres (1/15,000–25,000 population) and 3) satellite health posts (1/3,000–5,000 population). All three levels are supposed to be connected to each other by a referral system [15].

The HEWs deliver health-care services both at the health post and in the community, with a strong focus on sustained preventive health actions and increased health awareness; they provide antenatal care, and may attend deliveries, although whenever a complication emerges, they have to refer to the health centre, which is usually a walk of two to three hours. They are also in charge of supervising voluntary community health workers who are expected to support health education activities in the communities [17-19]. HEWs are accountable to the district health office for all the tasks of the HEP and they are also occasionally supervised and trained by midwives or nurses regarding maternal health activities. Most of the health centres in rural areas lack midwives because they work mainly at urban health centres and hospitals, and are the ones responsible for assisting women during labour.

Despite the strong efforts in spreading the HEP throughout the country, in terms of maternal health, universal access to services remains limited, particularly when it comes to skilled delivery attendance. In 2009, a survey conducted in four regions of Ethiopia reported that only 9% of women were giving birth at institutions [20]. Other recent studies in different regions of the country supported the finding that there is very little improvement in having more institutional deliveries [21-26]. Our previous study exploring women’s perspectives on maternal health-care services in Tigray showed evidence that women face a number of obstacles in accessing medical care services during delivery, including the ascendancy of elderly women, sociocultural beliefs, uncertain transportation and lack of trust in the quality of medical services [27].

HEWs and midwives are key human resources in the maternal health component of the HEP. Their insight into what is or is not working and the challenges they face during delivery care at health facilities should be considered highly relevant to understand the low number of institutional deliveries in the region and the country overall.

With help from ‘the three delays’ framework, this study explores health service providers’ perceptions of facilitators and barriers to the utilization of institutional delivery in Tigray, a northern region of Ethiopia.

## Methods

### Study area

Tigray has an estimated population of 4.3 million of which 51% are females. Eighty per cent of the population is estimated to live in rural areas and the majority of the inhabitants are Christian [28]. The region is divided into seven zones and 47 *weredas* (districts), of which 35 are rural and 12 urban. There is one specialized referral hospital as well as five zonal hospitals, seven district hospitals, 208 health centres and more than 600 *kebele*s (health posts) in the region. Maternal health-care coverage estimations from the Tigray Health Bureau indicate 75% for antenatal care, 20% for skilled delivery (those attended by nurses, midwives, health officers and/or physicians at health centres or hospitals, 13% for clean and safe deliveries (those attended by HEWs at home or health posts) and 90% for contraceptive use [29].

The study was conducted from September 2010 to January 2011 in two rural districts of the Tigray region, Ganta-afeshum and Kilte-awlaelo. These districts are located in the eastern zone of the region, 120 and 45 kilometres respectively from the regional capital Mekelle. In 2007, the total population of the two districts was estimated to be 188,384 inhabitants.

The two districts included in this study encompass 29 health posts with approximately 58 HEWs, 10 health centres and two hospitals. Five ambulances were available in the districts, two in Ganta-afeshum and three in Kilte-awlaelo. Data from the Tigray Health Bureau have estimated the antenatal care coverage in these two districts to be 53% and 80%, compared to the skilled (28% and 13% respectively) and clean/safe delivery attendance (21% and 9.5% respectively) [29].

### Participants

For this interview study, both HEWs and midwives were purposively selected with the aim of including informants with different training backgrounds. All of them had at least two years’ work experience at health-care facilities. A total of 12 interviewees participated: eight HEWs and four midwives employed at health posts and health centres/hospitals respectively, with an age range from 25 to 40 years old. The participants were all women, and differed in terms of educational level: HEWs were grade 10 students with one year training on primary health care; midwives were grade 12 students who had graduated with a diploma in nursing and had been employed at public health facilities for some years, then continued midwifery education (1.5 years) based on the national curriculum. In this study, users of the services were excluded from the interview, because an article about women’s experiences of delivery care was published recently by the authors [27].

### Data collection

A maternal and child health expert in the district health office identified potential participants and gave out their names and work addresses to the principal investigator (TG). The researcher visited the health facilities where the potential participants were working and requested their permission to be interviewed. In order to discuss topics more openly, the places chosen for the interviews were all private and comfortable for the participants. TG conducted all the interviews. Each in-depth interview lasted between 90 and 120 minutes.

At the beginning of the interview, the interviewer explained the general topic of the interview and encouraged the interviewee to express her ideas freely. The interview guide included semi-structured open-ended questions with certain key topics to be covered: reasons for women seeking/not seeking delivery care (DC), the role of elderly women, husbands and family members in decision-making processes, and encouraging and discouraging factors regarding giving birth at home or at a health facility (HF). Competency-related questions in regard to assisting births were raised based on the informants’ professional background. Relevant emerging issues were followed up in subsequent discussions during the interview.

All the interviews were conducted in Tigrigna, the mother tongue of the interviewer and the participants. The recorded interviews were transcribed and translated into English by final-year medical students and thoroughly double-checked against the original interview by the interviewer (TG). Handwritten notes were reviewed to find additional useful information.

### Data analysis

Data were analysed through a thematic analysis approach [30]. During the whole process of data collection and analysis, memos were recorded to capture ideas and reflections. The translated transcriptions were imported into Open Code software in order to manage the coding process [31]. Reading the material, the authors applied ‘the three delays model’ as a framework to guide the analysis. Consequently, as a first step the parts of the text that related to each delay were identified and marked distinctly as themes. As a second step, codes were developed for each of the themes and described as barriers or facilitators. Third, several codes were further refined with the aim of finding new information emerging and this served to fine-tune the labelling of the themes, making them closer to what the informants actually said.

### Ethical considerations

The study received ethical approval from the University of Mekelle, Research Ethical Review Committee of the College of Health Sciences, Northern Ethiopia. Permission was obtained from the district health authorities and written informed consent from every participant. Confidentiality and privacy were guaranteed: names and other information that would enable participants’ identification were removed. Participants were also informed that they could withdraw from participation at any time, for any reason and without negative consequences.

## Results

Three themes emerged from the analysis: the struggle between tradition and newly acquired knowledge, community willingness to deal with geographical barriers, and striving to do a good job with insufficient resources. These themes represent the three steps on the path toward receiving adequate institutional delivery care at a health facility. From each of these steps, facilitators and barriers were identified.

### The struggle between tradition and newly acquired knowledge

The participants felt that the awareness level of community members regarding the importance of institutional delivery has improved. Providing information during antenatal care check-ups or at home visits about the dangers of obstetric complications or the benefits of institutional delivery was described as a major facilitating factor for the perceived increase in the level of women’s awareness.

‘*Since we provide adequate advice about the benefit of health facility delivery during ANC, more women are coming to the health facility to give birth*’. (Midwife 7)

The relentless efforts of volunteer community health workers (VCHWs) and women’s health development groups were also highlighted as important for improving the utilization of institutional delivery. However, participants also described how the awareness differed among communities: those from rural areas are less attentive to the benefits of institutional delivery than urban communities.

The fact that HEWs live close to the health posts, engage in home visits and accompany women in labour who are referred to the hospital was cited as an opportunity to create close relationships and good interaction between the HEWs and the women.

‘*Since we have rooms for residence* [at the health post] *we are close and familiar to the community, which created a good relationship for us. As a result, women felt happy to be checked by us at the health post for ANC. They appreciated the proximity, our good approach and hospitality*’. (HEW 11)

Midwives reported a decrease in pregnant women seeking ANC at HCs, probably mirroring an increase in visits to health posts that are very close to their residences. However, both groups of participants agreed that the struggle to defeat barriers of institutional delivery was not an easy task. For instance, HEWs expressed that the majority of rural women prefer to give birth at home unless they are sick. Participants thought that perhaps this was because the community perceives delivery as a natural phenomenon that can be managed by the parturient and her companions.

‘*When we ask women the reason for not delivering at a health facility, their response is because they gave birth soon/fast after labour started at home. They also say* ‘*why should I come if I am not sick?*’ *They perceive that the health facility is only for sickness*’. (HEW 1)

HEWs also reported that the Tigrayan culture is full of traditional values that promote home delivery. The application of heat through a coal fire at home – not available at the health facilities – and the strong influence of elderly women in the decision-making process were examples mentioned by HEWs. Rituals performed at home in order to get Saint Mary’s support during delivery and the use of holy water sanctified by a priest were named as labour facilitators.

Participants mentioned that family members and relatives might prevent the woman from going to a health facility for delivery, since they perceive this moment as being of great susceptibility to catching the “evil eye”, with all the negative consequences associated with the latter, such as developing complications, failing to give birth or the death of the newborn or the mother.

‘*Traditionally, the family does not want to expose the issue of labour; it is like, if many people are informed about the issue, they associate it with some sort of devil/evil things[…] If the woman becomes very sick, eventually, they inform us, so HF is taken as a final option*’. (HEW 1)

### Community willingness to deal with geographical barriers

This theme describes the efforts of the community to facilitate the transportation of women to give birth at a health facility, and how geographical barriers, such as long distances, mountainous localities and bumpy roads, challenge these efforts.

‘*They are very kind if we ask them to help us carry the woman. The elderly ones escort us halfway and the youngsters carry her to our destination. Since the topography of the territory is challenging, community members are very much concerned for the safety of expectant mothers*’. (HEW 6)

Community members were recognized for their joint efforts in calling an ambulance for referral through the support of HEWs in the village. However, Participants noted that the use of mobile phones for seeking transport was hindered by poor network connections in the area.

Both midwives and HEWs expressed that despite the availability of HEWs, health posts in each sub-district and partial access to an ambulance, the difficult geographical setting of the localities, the scattered households and poor roads made referrals a lengthy and tiresome.

‘*The geographical setting of this locality is much too scattered. The topography is mountainous and steep. The main challenge is to carry a pregnant woman on steep roads. Moreover, the ambulance is not currently functioning*’. (HEW 5)

The interviewees also noticed that relatives of the women might feel uncomfortable asking non-relatives for voluntary services. Consequently, the number of relatives available in the location might decide the probability of transporting a woman to a health facility.

### Striving to do a good job with insufficient resources

This final theme describes the role of health service providers in attracting the community to modern health care along with the constraints of skilled human resources and the unfamiliar environment of the health facility.

All participants agreed that health providers in the hospital setting are better prepared to deal with deliveries and potential complications than health posts, which are mainly focused on promotion and prevention. Two main factors contributing to the increase in quality and attendance at hospital/health centre level were highlighted by the midwives: the presence of a gynaecologist (only at the hospital) and the availability of duty staff 24 hours a day and seven days a week.

‘*Women appreciate the free services they receive in the hospital, they like the hospital because it is always open for 24 hours, they are satisfied by the services offered; they acknowledge the final result of hospital delivery. They also appreciate the presence of skilled health professionals and the fact that a doctor can be consulted if required by circumstances*’. (Midwife 9)

While midwives did not deny the commitment of HEWs in ANC, family planning and other health preventive and promotion services, they were concerned about the low capability of HEWs in early detections of complications and in cases of referral.

Though the main activities of the HEWs are related to promotion and prevention, they noticed that the communities continuously demand basic curative services. HEWs revealed their disappointment at the absence of painkillers, iron and misoprostol tablets for preventing post-partum haemorrhage, as well as the equipment to do laboratory tests at the health post level. HEWs believed these factors negatively influenced the communities’ trust in their capacity and thus they prefer to seek care from health centres or hospitals instead of health posts. HEWs reflected on how they have been labelled as “people of latrines and kitchens”, a byname describing them as unqualified care providers. An additional factor that HEWs mentioned was the downgrading of clinics – previously staffed with nurses – to health posts.

‘*There is a feeling of discomfort in the community. They complain about having their health facility downgraded to a health post. The health post lacks medications like paracetamol* [painkillers] *and curative services for children are not being provided in the health post*’. (HEW 5)

HEWs were well aware of their limitations. They thought that the recent one-month clean and safe delivery care training was not adequate to assist labour and felt overburdened with the different components of the HEP. The fact that they have to spend a lot of time out in the field limited their availability to be at the health post attending women who may arrive for delivery.

The midwives and HEWs also mentioned some other aspects related to the quality of care and infrastructures. They acknowledged that not allowing family members to enter the delivery room at the hospital and certain negative provider attitudes could hinder institutional deliveries.

‘*They* [relatives of pregnant women] *complain that relatives are not allowed to enter the delivery room in the hospital; besides that, they complain that they are mistreated by some health professionals. I feel a woman shouldn’t be humiliated on top of the pain she suffers with*’. (HEW 2)

The absence of infection prevention materials such as masks, eye goggles and shoes, lack of electricity and shortage of water supply at health post levels were also major barriers mentioned by the HEWs.

‘*It is not smooth because there is a shortage of water and electrical facilities here. During delivery, darkness is challenging [since electricity is not available]. And we also have a shortage of materials for infection prevention.*’. (HEW 2)

## Discussion

This study has explored the perceived challenges of providing appropriate delivery care to women by HEWs and midwives in Tigray. Three main themes, closely connected with ‘the three delays model’, emerged describing the barriers to and facilitators of institutional delivery: the struggle between tradition and newly acquired knowledge, community willingness to deal with geographical barriers, and striving to do a good job with insufficient resources. The overall finding of this study is consistent with a previous study exploring women’s perspective, which indicated that women in this region were strongly influenced by both cultural factors that promoted home delivery and the perceived poor quality of the health system in terms of erratic emergency transportation and the unfriendly approach of health-care providers [27].

According to Thaddeus and Maine [7], the first step in preventing maternal deaths is reaching appropriate services able to deal with obstetric complications when they emerge and relates to making the decision to seek care at such services.

Providers interviewed in this study believe that women have received information on risks associated with home delivery during ANC. A previous study exploring women’s perceptions on maternal health care align with this finding, pointing out that women were aware of the complications associated with pregnancy and childbirth [27]. Providers emphasized the effort of the Women’s Health Development Group (a community-based women’s network spread all over the region) as a facilitator in our findings; this is consistent with a recent study, in which women’s group interventions resulted in a persuasive effect for mothers to use institutional delivery [32]. Furthermore, providing information about the benefits of delivery care during ANC has been proven to be effective by numerous studies [33-35].

However, there were other factors that acted as barriers in making the decision to seek institutional delivery. Some highly appreciated rituals, only available at home, were considered by the participants as keeping women from attending health-care facilities. These results are in agreement with previous studies from Ethiopia, where sociocultural characteristics, religion and family reasons can determine home deliveries [27,34,36-38]. Other studies from low- and middle-income countries are also consistent with our findings [12,39-41].

Another study from Ethiopia has also shown how women prefer home births because there they find the family support they need [26]. Other national and international studies also support this finding [34,35]. The childbirth humanization approach aims to increase cultural acceptability of services throughout the course of childbirth including aspects that are appreciated by communities and that might be beneficial during delivery – or at least not harmful: for example, from the presence of relatives, offering delivery in other positions, namely squatting, to getting used to being half undressed, or allowing the use of herbal teas. Some studies show evidence that making services more culturally acceptable for communities increases their use [42-45]. Intercultural training for health workers has also been suggested as a way to facilitate institutional delivery [44]. However, in our study, midwives and HEWs did not mention this possibility as a way of increasing women’s access to institutional delivery. The introduction of humanization approaches to delivery with a friendly environment in some districts of Tigray has recently been announced [46]: namely, allowing women to deliver in a position they prefer, including squatting, providing adequate soap and water to bathe after delivery, inviting husbands via a written letter to accompany their wives during labour and allowing relatives into the delivery room.

Other studies have highlighted that the decision to seek institutional delivery is not always in the hands of women, but rather rests with other relatives, mainly husbands, mothers and mothers-in-law [27]. Gender inequality can hinder a woman’s chance of making decisions in this regard. The lack of decision-making power of women in the provision of maternal health-care interventions has proven to be a challenge in many sub-Saharan African countries [5]. Gender discrimination and low levels of female education prevent women from seeking care, and accessing the best choices for themselves and their children’s health [5,19]. However, in this study, participants did not mention gender inequality as a barrier to deciding to seek institutional delivery; this might reflect that HEWs and nurses, despite being women and from similar backgrounds to the patients they serve, are not aware of gender inequality dynamics that affect women’s decision-making.

The second step in preventing maternal deaths relates to accessibility issues, namely reaching services in time to adequately manage obstetric complications. Challenges of inaccessibility of health facilities were pointed out in this study. Despite the willingness of the community to solve problems of transportation, it was tough to refer women from hard-to-reach areas to higher-level health facilities for delivery. Several national and international studies are in line with this finding [12,34,47-49]. For instance, a review by Gabrysch et al. reported that distance from health facilities and poor road conditions are major constraints of institutional delivery [47]. Despite the fact that mobile phones could help to speed up the transport during emergencies, a study from the same region reported that the challenge to integrating mobile health applications into the Ethiopian primary health-care system was due to the poor network coverage [50]. Similarly, our study emphasized the poor network connections as barriers to institutional delivery.

The Tigray Regional Health Bureau is developing strategies to overcome the accessibility barriers. Recently a report from the health bureau indicated that a number of actions were being implemented: for example, rural youth members are organized under the theme of “No woman should die while giving life” to carry labouring mothers using a local stretcher to a nearby health centre, and selected health centres are providing waiting rooms and beds for mothers approaching their expected date of delivery and residing in hard-to-reach areas.

Despite the current commitment of the Tigray Regional Health Bureau, the problem of transportation remains a key challenge in many remote areas. Strong links among HEWs, Kebele administration delegates, women leaders and ambulance drivers are crucial. Appointing health facility community members has been shown to play an important role in representing community interests and helping to oversee facility management [51].

The last step in preventing maternal deaths consists of ensuring that women actually get adequate services once they reach the health facility, namely services with enough material and human resources to deal with obstetric complications. In this study, participants indicated that the hospital setting was a better place for delivery than home births: services are free of charge, available 24 hours a day, and professionals working at the hospital are more competent.

While participants perceived hospitals as highly appreciated, HEWs’ skills and competences were distrusted. Midwives pointed out HEWs’ lower competency in identifying high-risk pregnant women for referral, incomplete history taking and referring women without a referral slip. Despite the fact that the commitment of HEWs is undeniable, the gap in their knowledge and skills was emphasized in our study. This finding was similar to a study conducted in the Western Tigray region that underlines shortcomings to be corrected [52].

The HEP has put an emphasis on HEWs as the first health professionals that the community members will contact; they should be the entry point to the health-care system [16,17]. According to the HEP, HEWs should assist deliveries when mothers are giving sudden birth at home. During such home deliveries, HEWs are also responsible for facilitating transportation for women who need referral [16,43]. This study highlights several limitations that HEWs face in actually fulfilling those tasks. First, the HEWs felt ill prepared to deal with deliveries. The training they receive and the multiple tasks they should perform (often outside the health post) under the HEP limited their performance. This finding is consistent with a study by Koblinsky et al. in which the reason for a low level of institutional delivery prevalence was reported to be due to the engagement of little work time (average of two hours per week) for delivery services [19]. Another study from southern Ethiopia reports similar results to our findings and concludes that HEWs devote more than 70% of their time to home visits alone [53]. To balance this situation, the Tigray Regional Health Bureau is currently, through outreach services, engaging midwives and nurses in the provision of PMTCT (prevention of HIV transmission from maternal to child), ANC and post-natal care to women who reside in hard-to-reach areas [43].

Second, HEWs felt that the community did not trust their professional skills. The downgrading of health posts, which were previously staffed with nurses, was considered to be disappointing for communities, put pressure on HEWs to continue performing curative services and resulted in HEWs being worried and frustrated due to their inability to adequately meet the expectations of the community. To reduce the unmet expectation of the community, the Tigray Regional Health Bureau is currently providing oral antibiotics for the treatment of acute respiratory infections and oral rehydration salts for diarrhoeal diseases to children under five at the health post level.

Third, participants expressed their discomfort at working in health facilities where necessary drugs and supplies were seldom available, and where working conditions were poor, namely, there was no electricity or water supply. The midterm review of Health Sector Development Programme (HSDP) III that was conducted in 2010 reinforces, at a national level, unmet community expectation (pointed out in our findings): “though the health service coverage is nearly 100% (due to the availability of HEWs and health posts), the utilization rate per person per year was only 0.32 while the target is 0.66” [16]. Furthermore, our result is supported by a study from Malawi, where the lack of material resources was reported to have a big impact on the acceptability of maternal health services [54].

Fourth, HEWs felt that professionals at health centres and hospitals did not trust the skills of the HEWs, and thus they treated them unkindly when referring patients. This could further hinder the development of a strong referral network between health posts and more specialized services. Our findings also pointed out the poor capability of nurses and midwives in referring high-risk pregnant women to high-level health facilities. This was also observed in an Indonesian study, where late and poor-quality referral was reportedly due to the poor performance of health staff [9]. Similarly, another study from Laos highlighted the lack of antenatal care equipment and hence the failure of health workers to identify serious conditions such as pre-eclampsia [55]. Resource-limited countries like Ethiopia tend to train less skilled health workers, thus contributing to the low prevalence of institutional delivery. This poses a question regarding the training provided to Ethiopian midwives, something that might not meet the criteria for educational competency model curriculum promoted by the International Confederation of Midwives [56].

### Limitations

It would have been interesting to include more participants with different perspectives to have a more comprehensive picture. However, we have recently published a paper exploring women’s perception of maternal health care in Tigray [27]. In the discussion, findings from this previous study with women in the area have been used to contrast the findings from this study.

Although interviewees seemed to participate actively and freely during the interview, the setting of the interviews where midwives and HEWs were interviewed, and the first author's position as a member of the university's medical faculty and as a former staff member of the Regional Health Bureau interviewing health workers, could have affected the results through social desirability bias.

Participants were purposely selected for their ability to contribute to the research question, and an effort was made to contextualize the results and to detail the analytic process. However, the way informants were selected by maternal and child health experts could have led to social desirability bias too, namely respondents providing a more positive attitude towards institutional delivery.

Despite these limitations, measures were taken to strengthen trustworthiness. In order to enhance dependability, an emergent design was followed and the question guide incorporated relevant issues that emerged from previous interviews. Since the first author was originally from Tigray and living in the area, credibility – how well the findings had captured the reality being explored–was enhanced by prolonged engagement. Credibility was also enhanced due to the fact that the other researchers were not familiar with the setting contributing to adding the external perspective.

## Conclusions

The participants in this study emphasized the major barriers to institutional delivery that are closely connected with ‘the three delays model’. The struggle between tradition and newly acquired knowledge, geographical barriers and insufficient resources at health facilities, particularly the lack of the ability of Health Extension Workers to provide delivery services, were pointed out.

Nurses and HEWs are expected to provide maternal health care with as much close contact with mothers as possible. In reality, the lack of drugs and equipment, poor infrastructure and insufficient expertise (in terms of HEWs) hindered their performance. These factors together with transportation barriers made women’s access to skilled delivery and emergency obstetric and neonatal care difficult.

Despite the initiatives being implemented by the Tigray Regional Health Bureau, much still needs to be done to humanize delivery care at a broader level in the region. The major issue of geographical inaccessibility needs to be addressed quickly.

The poor capacity of HEWs to provide delivery services calls for a reconsideration of the staffing patterns of remote health posts. Readdressing the issue of downgraded health facilities would address unmet community needs.

## Competing interests

The authors declare that they have no competing interests.

## Authors’ contributions

TG contributed to the conception, design, data collection, analysis, interpretation, writing up and preparing the draft of the manuscript. MSS and IG contributed to the conception, providing scientific advices on the design of the study, data analysis and throughout the preparation of the manuscript. KE contributed in revising the interpretation and throughout the preparation of the manuscript. All authors read and approved the final manuscript.

## Pre-publication history

The pre-publication history for this paper can be accessed here:

http://www.biomedcentral.com/1471-2393/14/137/prepub
